# Supplementary material for: Comparative Analysis Identifies Similarities between the Human and Murine Microglial Sensomes
Source: Int J Mol Sci. 2021 Feb 2;22(3):1495. doi: 10.3390/ijms22031495 (PMC7867338; doi:10.3390/ijms22031495)
Supplement: Supplementary file 1 [file ijms-22-01495-s001.pdf]

Supplementary Materials

Figure S1

A

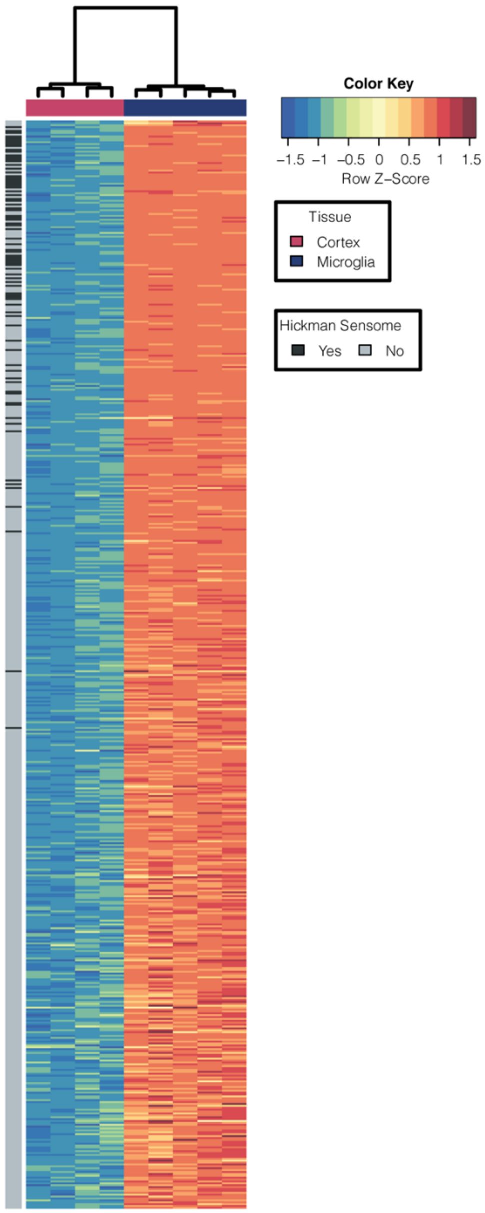

Supplemental Figure S1. Distinct difference in expression of 576 sensome genes comparing cortex versus microglia. (**A**) This heatmap shows all 576 sensome candidate genes ordered by DE and with the left column shows if the gene is present in the "Hickman et al. sensome"

Figure S2

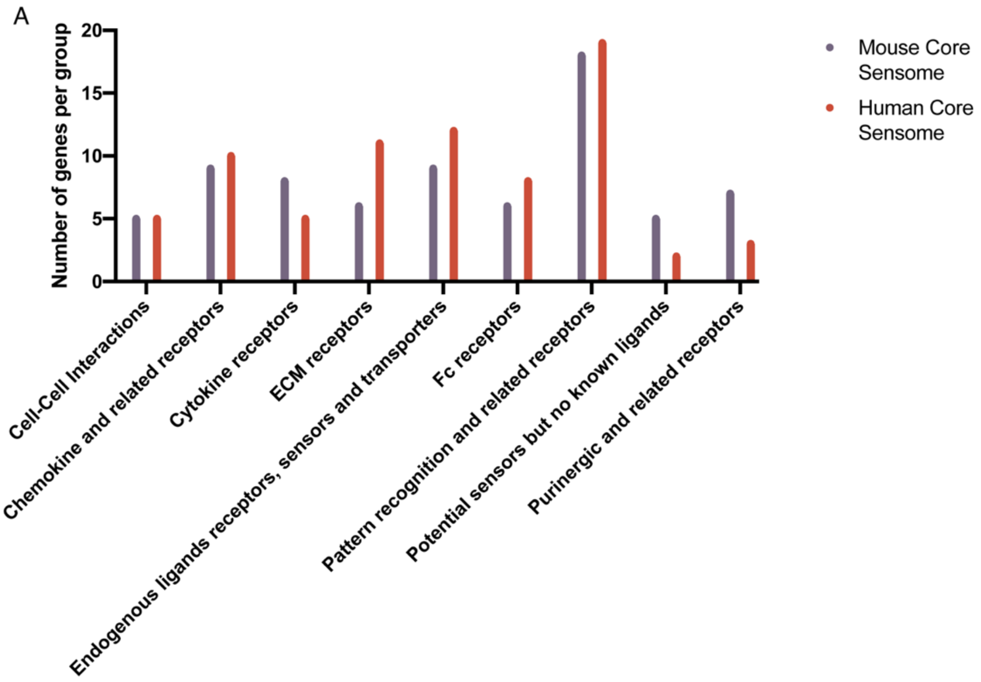

Supplemental Figure S2. Mouse sensome and human sensome genes categorized by group. (**A**) Bar graph showing the number of mouse and human sensome genes per group (Cell-Cell Interactions, Chemokine and related receptors, Cytokine receptors, ECM receptors, Endogenous ligands receptors, sensors and transporters, Fc receptors, Pattern recognition and related receptors, Potential sensors but no known ligands and Purinergic and related receptors).

Figure S3

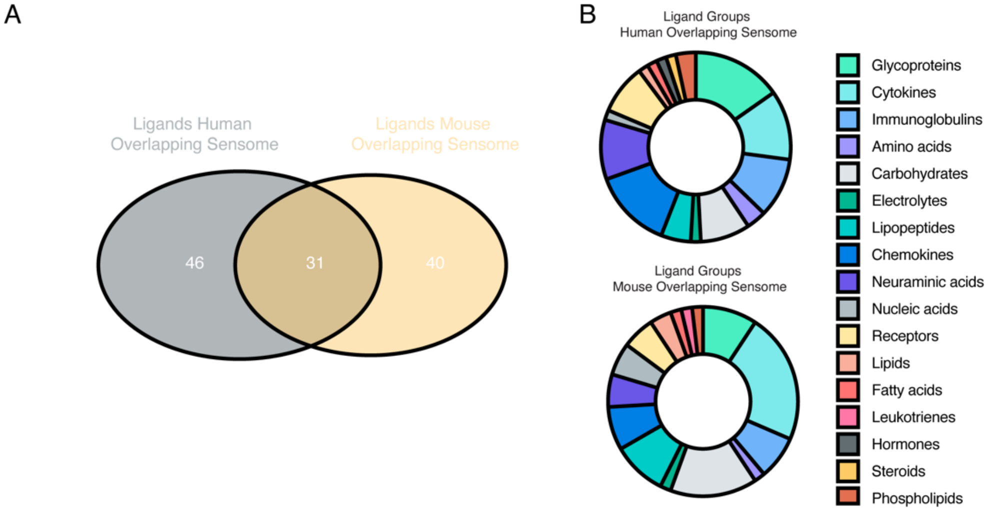

Supplementary Figure S3. Overlap of ligands recognized by microglia sensome (**A**) Overlap between the ligands of the receptors from respectively human and mouse core sensome was shown using Venn Diagrams. (**B**) Ligands of human and mouse receptors categorized in groups (Glycoproteins, Cytokines, Immunoglobulin, Amino acids, Carbohydrates, Electrolytes, Lipopeptides, Chemokines, Neuraminic acids, Nucleic acids, Receptors, Lipids, Fatty acids, Leukotrienes, Hormones, Steroids and Phospholipids) and spread of different groups shown as parts of whole again highlighting that the distribution of ligands what the human and mouse sensome genes can sense (Categorization of ligands in **Supplementary Table S1**).

Figure S4

Aging

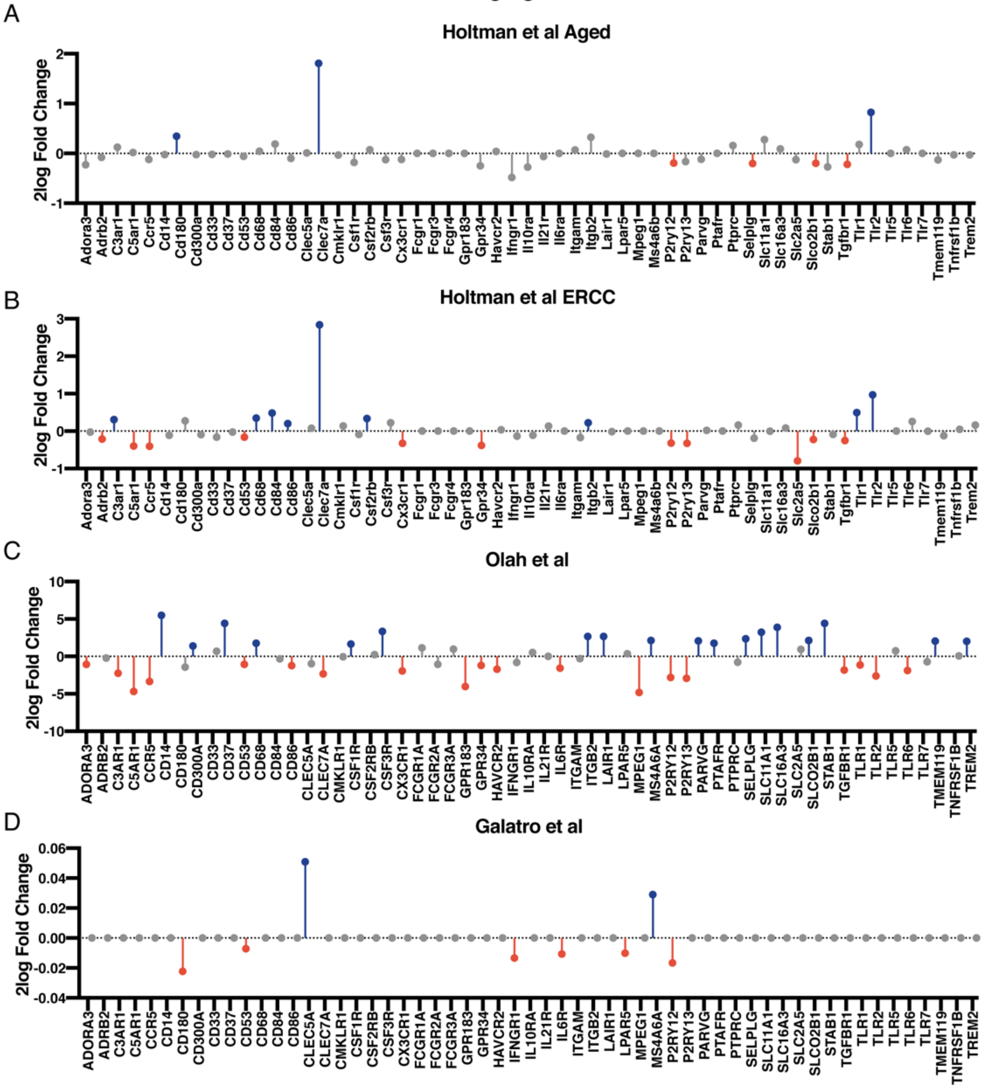

Supplementary Figure S4. Microglia core sensome expression during aging. (**A**) Two-log fold change of microglia core sensome genes in aging mice derived from Holtman et al. [12]. (**B**) Accelerated aging model (ERCC1), with impaired DNA repair mechanism, shows changes of microglia core sensome expression [12]. (**C**) Microglia core sensome expression during aging in human derived from Olah et al. [10]. (**D**) Gene expression data extracted from Galatro et al. showing the expression of microglia core sensome gene[15]. Red bars display showing gene significantly upregulated, blue bars represent gene significantly downregulated (Detailed expression data in **Supplementary Table S5**).

Figure S5

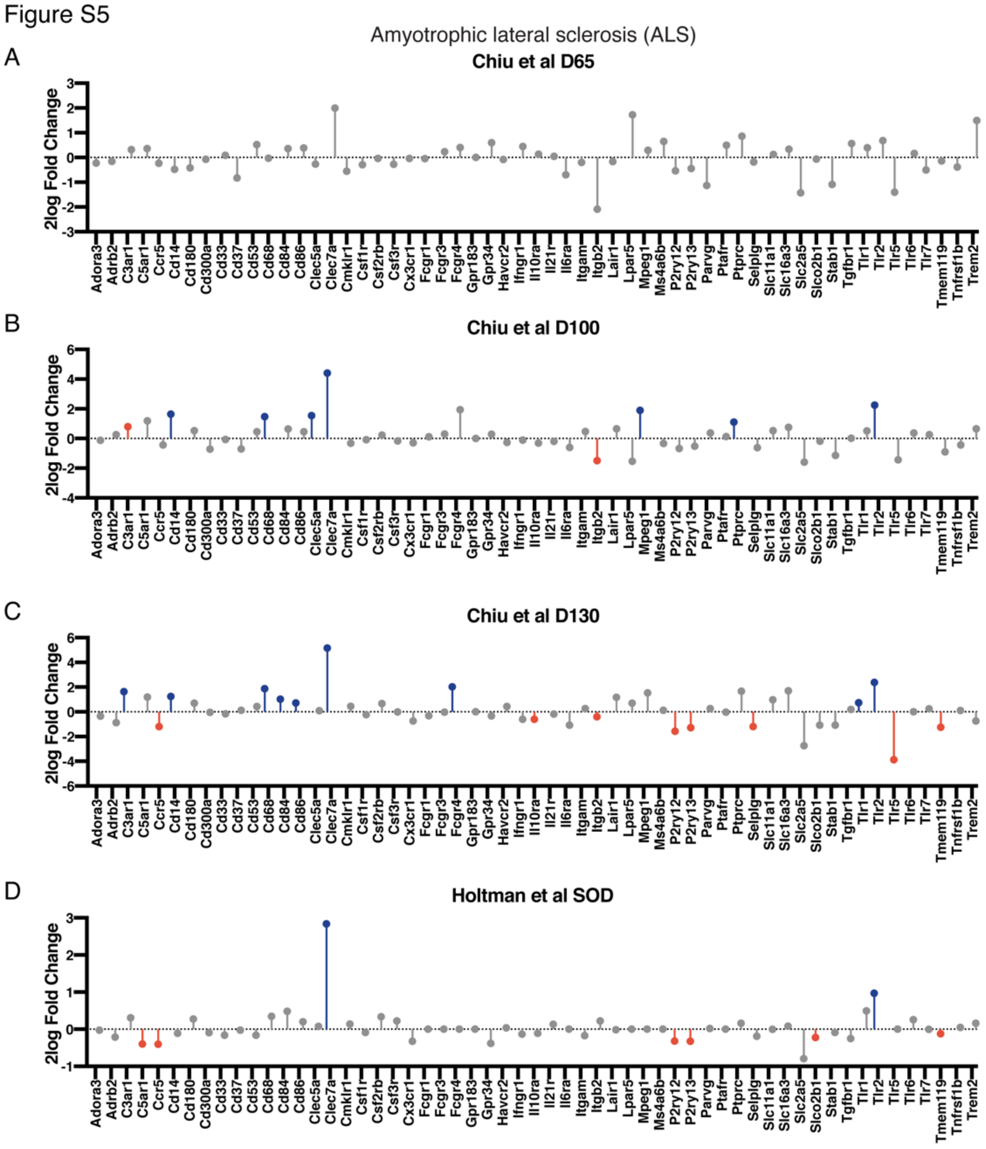

Supplementary Figure S5. Microglia core sensome expression in ALS. (**A**) Microglia core sensome expression during ALS disease progression (A) 65 days, (**B**) 100 days and at the end of disease (**C**) (130 days) after onset as analyzed by Chiu et al. [11]. (**D**) Microglia gene expression in control versus ALS mouse model (SOD1) derived from Holtman et al. [12]. Red bars display showing gene significantly upregulated, blue bars represent gene significantly downregulated (Detailed expression data in **Supplementary Table S5**).

Figure S6

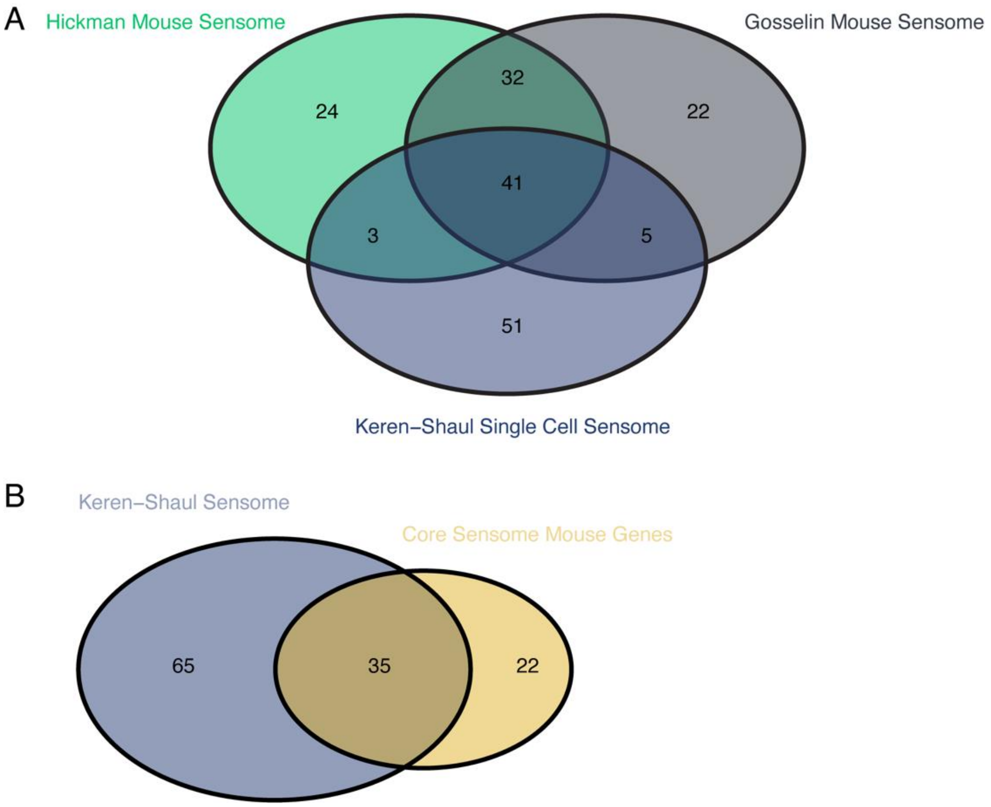

Supplemental Figure S6. Overlap of microglia core sensome in single cell microglia dataset. (**A**) Overlap between the microglia sensome extracted from bulk RNAseq data and a validation sensome extracted from single cell homeostatic microglia data. (**B**) Overlap between microglia core sensome and manually curated single cell sensome where genes were included as described in methods and material filtered from the highest average UMI from homeostatic [4].

Supplementary Table S1. Mouse and human sensome genes with ligands recognized by the receptors.

**Ligand group**	**Ligand**	**Mouse core sensome**	**Human core sensome**
amino acids	chondroitin sulfate moiety of glycosaminoglycan chains		ADGRE2
amino acids	neutral amino acids		SLC1A5
amino acids	Amino acids	Slc7a7	
carbohydrates	lectins, selectins	Cd68	CD68
carbohydrates	carbohydrate	Clec5a	CLEC5A
carbohydrates	carbohydrates	Clec7a	CLEC7A
carbohydrates	ADP	P2ry12	P2RY12
carbohydrates	ADP	P2ry13	P2RY13
carbohydrates	fructose	Slc2a5	SLC2A5
carbohydrates	carbohydrates		MRC1

carbohydrates	carbohydrate	Clec4a3	
carbohydrates	UTP	P2ry6	
carbohydrates	cyclic dinucleotides, cyclic di-GMP, GMP-AMP	Tmem173	
chemokines	C3A	C3ar1	C3AR1
chemokines	C5A	C5ar1	C5AR1
chemokines	CCL3/MIP-1-alpha, CCL4/MIP-1-beta and RANTES	Ccr5	CCR5
chemokines	adipokine chemerin/RARRES2 and for the omega-3 fatty acid derived molecule resolvin E1	Cmklr1	CMKLR1
chemokines	Fractalkine	Cx3cr1	CX3CR1
chemokines	MIP-1-alpha, MIP-1-delta, RANTES, and MCP-3		CCR1
chemokines	FMLP		FPR1
chemokines	C3b cleavage product of C3		VSIG4
chemokines	C5a	C5ar2	
cytokines	CSF1 and IL34	Csf1r	CSF1R
cytokines	interleukin-3, interleukin-5 and granulocyte-macrophage colony-stimulating factor	Csf2rb2	CSF2RB
cytokines	Colony-stimulating factor	Csf3r	CSF3R
cytokines	Inf-γ	Ifngr1	IFNGR1
cytokines	IL10	Il10ra	IL10RA
cytokines	IL-21	Il21r	IL21R
cytokines	IL-6	Il6ra	IL6R
cytokines	platelet activating factor	Ptafr	PTAFR
cytokines	TGFB1, TGFB2 and TGFB3	Tgfbr1	TGFBR1
cytokines	TNFSF2/TNF-alpha	Tnfrsf1b	TNFRSF1B
cytokines	Latency-associated peptide (LAP)		NRROS
cytokines	CCL19 and chemerin/RARRES2	Ccrl2	
electrolytes	iron and manganese	Slc11a1	SLC11A1
fatty acids	oxidized free fatty acids		GPR132
fatty acids	medium-chain free fatty acid (FFA)	Gpr84	
glycoproteins	fibrinogen, factor X and ICAM1	Itgam	ITGAM
glycoproteins	DPP4	Ptprc	PTPRC
glycoproteins	E- and P-selectins	Selplg	SELPLG
glycoproteins	glycans with terminal alpha-linked mannose or fucose residues		CLEC17A
glycoproteins	fibrinogen		ITGAX

glycoproteins	cytotactin, fibronectin, laminin, matrix metalloproteinase 2, osteopontin, osteomodulin, prothrombin, thrombospondin, vitronectin and von Willebrand factor		ITGB3
glycoproteins	class I MHC antigens		LILRB1
glycoproteins	class I MHC antigen		LILRB4
glycoproteins	HIV-1 gp41		TLR10
glycoproteins	small molecular motifs named pathogen-associated molecular pattern (PAMPs)		TLR5
glycoproteins	fibronectin	Itgb5	
glycoproteins	FGL1, MHC-II	Lag3	
glycoproteins	E- and P-selectins	Selplg	
hormones	epinephrine	Adrb2	ADRB2
immunoglobulins	Fc region of immunoglobulins gamma	Fcgr1	FCGR1A
immunoglobulins	Fc region of immunoglobulins gamma	Fcgr3	FCGR2A
immunoglobulins	Fc region of IgG	Fcgr4	FCGR3A
immunoglobulins	cleaved immunoglobulin generated by microbial pathogens		LILRA2
immunoglobulins	immunoglobulin		MILR1
immunoglobulins	IgE	Fcer1g	
leukotrienes	cysteinyl leukotrienes	Cysltr1	
lipids	amyloid-beta protein 42, lipoprotein particles such as LDL, VLDL, and HDL and for apolipoproteins such as APOA1, APOA2, APOB, APOE, APOE2, APOE3, APOE4, and CLU. Binds phospholipids (preferably anionic lipids) such as phosphatidylserine, phosphatidylethanolamine, phosphatidylglycerol and sphingomyelin	Trem2	TREM2
lipids	oleoyl-L-alpha-lysophosphatidic acid (LPA)		LPAR6
lipopeptide	acetylated low density lipoprotein	Stab1	STAB1
lipopeptide	oxLDL		OLR1
lipopeptide	LPS, free fatty acids, electronegative LDL (LDL-)	Tlr4	
lipopeptides	bacterial lipopolysaccharide	Cd14	CD14
lipopeptides	LPS	Cd180	CD180

lipopeptides	diacylated and triacylated lipopeptidess	Tlr1	TLR1
lipopeptides	mycoplasmal macrophage-activating lipopeptides-2kD (MALP-2), soluble tuberculosis factor (STF), phenol-soluble modulin (PSM) and B. burgdorferi outer surface protein A lipoprotein (OspA-L)	Tlr2	TLR2
lipopeptides	diacylated and, to a lesser extent, triacylated lipopeptidess	Tlr6	TLR6
miscellaneous/unknown	Flagellin	Tlr5	TLR5
miscellaneous/unknown	Unknown	Cd37	CD37
miscellaneous/unknown	Unknown	Cd53	CD53
miscellaneous/unknown	Oxysterol	Gpr183	GPR183
miscellaneous/unknown	Unknown	Gpr34	GPR34
miscellaneous/unknown	collagen	Lair1	LAIR1
miscellaneous/unknown	Unknown	Mpeg1	MPEG1
miscellaneous/unknown	Unknown	Ms4a6b	MS4A6A
miscellaneous/unknown	Unknown	Parvg	PARVG
miscellaneous/unknown	lactate	Slc16a3	SLC16A3
miscellaneous/unknown	Unknown	Tmem119	TMEM119
miscellaneous/unknown	folate		FOLR2
miscellaneous/unknown	Hyaluronic acid		LYVE1
miscellaneous/unknown	Unknown		MS4A6A
miscellaneous/unknown	Unknown		MS4A7
miscellaneous/unknown	Unknown		NFAM1
miscellaneous/unknown	prostaglandin E2 (PGE2)		PTGER4
miscellaneous/unknown	succinate		SUCNR1
miscellaneous/unknown	Unknown		TMEM156
miscellaneous/unknown	Unknown	Cmtm7	
miscellaneous/unknown	Unknown	Ecscr	
miscellaneous/unknown	Unknown	Entpd1	
miscellaneous/unknown	Unknown	Fcrl1	
miscellaneous/unknown	Unknown	I830077J02Rik	
miscellaneous/unknown	Unknown	Slamf9	
miscellaneous/unknown	Unknown	Tlr12	
miscellaneous/unknown	Unknown	Upk1b	
miscellaneous/unknown	Unknown	Vsir	
neuraminic acids	Lectin / Sialic acid	Cd33	CD33
neuraminic acids	Lectin / Sialic acid		SIGLEC10

neuraminic acids	Lectin / Sialic acid		SIGLEC11
neuraminic acids	Lectin / Sialic acid		SIGLEC14
nucleic acids	adenosine	Adora3	ADORA3
nucleic acids	uridine-containing single strand RNAs (ssRNAs) of viral origin or guanosine analogs	Tlr7	TLR7
nucleic acids	23S rRNA	Tlr13	
phospholipids	phosphatidylethanolamine (PE) and phosphatidylserine (PS)	Cd300a	CD300A
phospholipids	LGALS9, PtSer	Havcr2	HAVCR2
phospholipids	Lysophosphatidic acid (LPA)	Lpar5	LPAR5
receptors	CD84	Cd84	CD84
receptors	CD28 or CTLA-4	Cd86	CD86
receptors	CD11a, CD11b, CD11c and CD11d. ICAM1, ICAM2, ICAM3 and ICAM4	Itgb2	ITGB2
receptors	ICAM 1-4		ITGAL
receptors	ICAM 1		SPN
steroids	estrone–3-sulfate	Slco2b1	SLCO2B1

Supplementary Table S2. List of genes and overlap between Mouse Sensome Core, Mouse Sensome Extended, Hickman Sensome and Gosselin Sensome (● = overlap, ○ = no overlap).

Mouse Sensome Extended	Hickmann Sensome	Gosselin Sensome	Mouse Sensome Core
Itgb5	●	●	●
Ccrl2	●	●	●
Ifngr1	●	●	●
Ecscr	●	●	●
Fcer1g	●	●	●
Slc7a7	●	●	●
Icam1	●	●	●
Cd68	●	●	●
Lag3	●	●	●
Clec5a	●	●	●
Trem2	●	●	●
Entpd1	●	●	●
Fcgr3	●	●	●
Tgfbr1	●	●	●
I830077J02Rik	●	●	●
Clec4a3	●	●	●
Cmtm7	●	●	●
Itgam	●	●	●
Lair1	●	●	●
Adgre1	●	●	●
Tlr4	●	●	●
Fcgr2b	●	●	●
Csf2rb2	●	●	●
Cd53	●	●	●
Tlr12	●	●	●
Cmklr1	●	●	●
Fcrl1	●	●	●
Siglech	●	●	●
Adora3	●	●	●
Cd86	●	●	●
Vsir	●	●	●
Cd37	●	●	●
Ptafr	●	●	●
Gpr183	●	●	●
Il6ra	●	●	●
Fcgr1	●	●	●

Csf1r	●	●	●
Havcr2	●	●	●
P2ry6	●	●	●
Upk1b	●	●	●
Itgb2	●	●	●
Cd33	●	●	●
Tmem119	●	●	●
C5ar2	●	●	●
Tnfrsf1b	●	●	●
Tmem173	●	●	●
Tlr6	●	●	●
Gpr34	●	●	●
Slco2b1	●	●	●
Il21r	●	●	●
Cd84	●	●	●
Selplg	●	●	●
Slamf9	●	●	●
Slc2a5	●	●	●
Ptprc	●	●	●
P2ry12	●	●	●
Siglece	●	●	●
C3ar1	●	●	●
C5ar1	●	●	●
Gpr84	●	●	●
P2ry13	●	●	●
Cd180	●	●	●
Cd14	●	●	●
Cx3cr1	●	●	●
Il10ra	●	●	●
Tlr7	●	●	●
Ccr5	●	●	●
Cd48	●	●	●
Tlr13	●	●	●
Cysltr1	●	●	●
Lpar5	●	●	●
Tlr1	●	●	●
Tlr2	●	●	●
A630033H20Rik	●	○	○
Tgfbr2	●	○	○

Tnfrsf17	●	○	○
Cmtm6	●	○	○
Fcgr4	●	○	○
Ly86	●	○	○
Clec7a	●	○	○
Cxcl16	●	○	○
Ltf	●	○	○
Cd74	●	○	○
Pilra	●	○	○
Ifitm6	●	○	○
Lgals9	●	○	○
Tmem37	●	○	○
Cd52	●	○	○
Cd79b	●	○	○
Slc16a3	●	○	○
Icam4	●	○	○
Tyrobp	●	○	○
Tnfrsf13b	●	○	○
Cd22	●	○	○
Clec4a2	●	○	○
Clec4b1	●	○	○
Lilra5	●	○	○
Tmem8c	●	○	○
Gpr160	●	○	○
Cd101	●	○	○
Csf3r	○	●	○
Tlr9	○	●	○
Slc11a1	○	●	○
Ly9	○	●	○
H2-Ob	○	●	○
Abcc3	○	●	○
Tlr5	○	●	○
Adrb2	○	●	○
Cnr2	○	●	○
Cd300a	○	●	○
Il7r	○	●	○
Csf2rb	○	●	○
1810011H11Rik	○	●	○
Cd300c2	○	●	○

Tmigd3	○	●	○
Mpeg1	○	●	○
Lcp1	○	●	○
Il1rl2	○	●	○
Ms4a6b	○	●	○
F11r	○	●	○
Ticam2	○	●	○
Stab1	○	●	○
Ms4a6d	○	●	○
H2-DMb1	○	●	○
Tnfrsf14	○	●	○
Parvg	○	●	○
Slc15a3	○	●	○

Supplementary Table S3. List of genes and overlap between Human Sensome Core, Human Sensome Extended, Gosselin Sensome and Galatro Sensome (● = overlap, ○ = no overlap).

**Human Sensome Extended**	**Gosselin Sensome**	**Galatro Sensome**	**Human Sensome Core**
TMEM119	●	●	●
GPR132	●	●	●
CD33	●	●	●
TNFRSF1B	●	●	●
LPAR5	●	●	●
LILRA2	●	●	●
SLC16A3	●	●	●
P2RY12	●	●	●
CD300A	●	●	●
C5AR1	●	●	●
GPR183	●	●	●
MILR1	●	●	●
FOLR2	●	●	●
OLR1	●	●	●
CLEC17A	●	●	●
SLC1A5	●	●	●
NFAM1	●	●	●
CX3CR1	●	●	●
CSF3R	●	●	●
P2RY13	●	●	●
LYVE1	●	●	●
ITGB3	●	●	●
TMEM156	●	●	●
ITGB2	●	●	●
ADRB2	●	●	●
ITGAL	●	●	●
FPR1	●	●	●
PARVG	●	●	●
SUCNR1	●	●	●
ITGAM	●	●	●
NRROS	●	●	●
SPN	●	●	●
SLCO2B1	●	●	●
SIGLEC11	●	●	●
LILRB4	●	●	●

MPEG1	●	●	●
FCGR2A	●	●	●
FCGR1B	●	●	●
CD86	●	●	●
CSF2RB	●	●	●
FCGR3A	●	●	●
PTAFR	●	●	●
IL10RA	●	●	●
LPAR6	●	●	●
LAIR1	●	●	●
ITGAX	●	●	●
CSF1R	●	●	●
SLC2A5	●	●	●
SIGLEC10	●	●	●
SIGLEC8	●	●	●
FCGR1A	●	●	●
LILRB1	●	●	●
ADGRE2	●	●	●
C3AR1	●	●	●
SIGLEC14	●	●	●
CD14	●	●	●
MS4A7	●	●	●
SIGLEC9	●	●	●
CCR1	●	●	●
TLR7	●	●	●
TLR10	●	●	●
CLEC7A	●	●	●
CCR5	●	●	●
VSIG4	●	●	●
CMKLR1	●	●	●
STAB1	●	●	●
TLR6	●	●	●
PTPRC	●	●	●
PTGER4	●	●	●
TLR5	●	●	●
MRC1	●	●	●
MS4A6A	●	●	●
SLC11A1	●	●	●
CD84	●	●	●

TLR2	●	●	●
TLR1	●	○	○
IL21R	●	○	○
PLXDC2	●	○	○
TREML1	●	○	○
LY75	●	○	○
TLR3	●	○	○
MSR1	●	○	○
GPR34	●	○	○
GPR65	●	○	○
TLR8	●	○	○
TGFBR1	●	○	○
CD180	●	○	○
MS4A4A	●	○	○
IGSF6	●	○	○
IL6R	●	○	○
RHBDF2	●	○	○
HAVCR2	●	○	○
CD274	●	○	○
TNFRSF11A	●	○	○
CD53	●	○	○
ADORA3	●	○	○
IFNGR1	●	○	○
MERTK	●	○	○
TGFBR2	●	○	○
SUSD6	●	○	○
ADGRG5	○	●	○
CD300LF	○	●	○
CEACAM1	○	●	○
IL1R2	○	●	○
SELPLG	○	●	○
CD300E	○	●	○
LILRB2	○	●	○
FCN1	○	●	○
TNFRSF13C	○	●	○
APOBR	○	●	○
LILRA4	○	●	○
CD37	○	●	○
LILRA6	○	●	○

CSF2RA	○	●	○
LILRB3	○	●	○
CLEC5A	○	●	○
FCGR3B	○	●	○
FCGR2C	○	●	○
LILRA1	○	●	○
CD69	○	●	○
CLEC9A	○	●	○
TREM2	○	●	○
CD177	○	●	○
CD74	○	●	○
CD68	○	●	○

Supplementary Table S4. Overlap of human and mouse sensome revealed the microglia core sensome

Mouse to human core overlap	Human to mouse core overlap
ADORA3	Adora3
ADRB2	Adrb2
C3AR1	C3ar1
C5AR1	C5ar1
CCR5	Ccr5
CD14	Cd14
CD180	Cd180
CD300A	Cd300a
CD33	Cd33
CD37	Cd37
CD53	Cd53
CD68	Cd68
CD84	Cd84
CD86	Cd86
CLEC5A	Clec5a
CLEC7A	Clec7a
CMKLR1	Cmklr1
CSF1R	Csf1r
CSF2RB	Csf2rb
CSF3R	Csf3r
CX3CR1	Cx3cr1
FCGR1A	Fcgr1
FCGR2A	Fcgr3
FCGR3A	Fcgr4
GPR183	Gpr183
GPR34	Gpr34
HAVCR2	Havcr2
IFNGR1	Ifngr1
IL10RA	Il10ra
IL21R	Il21r
IL6R	Il6ra
ITGAM	Itgam
ITGB2	Itgb2
LAIR1	Lair1
LPAR5	Lpar5
MPEG1	Mpeg1
P2RY12	P2ry12

P2RY13	P2ry13
PARVG	Parvg
PTAFR	Ptafr
PTPRC	Ptprc
SELPLG	Selplg
SLC11A1	Slc11a1
SLC16A3	Slc16a3
SLC2A5	Slc2a5
SLCO2B1	Slco2b1
STAB1	Stab1
TGFBR1	Tgfbr1
TLR1	Tlr1
TLR2	Tlr2
TLR5	Tlr5
TLR6	Tlr6
TLR7	Tlr7
TMEM119	Tmem119
TNFRSF1B	Tnfrsf1b
TREM2	Trem2

Supplemental table S5. Microglia core sensome gene expression in multiple mouse models and human disease

**Chiu et al. D65**				
Gene name	Log2 fold change	Fold-change.Mutant/Control	P-value	FDR
Adora3	-0.227	0.854	0.197	0.690
Adrb2	-0.155	0.898	0.481	0.836
C3ar1	0.315	1.244	0.125	0.622
C5ar1	0.362	1.285	0.424	0.810
Ccr5	-0.234	0.850	0.330	0.762
Cd14	-0.475	0.719	0.005	0.344
Cd180	-0.417	0.749	0.008	0.405
Cd300a	-0.072	0.951	0.907	0.975
Cd33	0.088	1.063	0.633	0.889
Cd37	-0.823	0.565	0.003	0.305
Cd53	0.520	1.434	0.214	0.701
Cd68	-0.032	0.978	0.852	0.961

Cd84	0.359	1.283	0.230	0.705
Cd86	0.383	1.304	0.113	0.613
Clec5a	-0.263	0.833	0.401	0.795
Clec7a	1.998	3.995	0.102	0.598
Cmklr1	-0.555	0.681	0.210	0.700
Csf1r	-0.293	0.816	0.225	0.705
Csf2rb	-0.035	0.976	0.785	0.941
Csf3r	-0.279	0.824	0.208	0.700
Cx3cr1	-0.039	0.973	0.722	0.920
Fcgr1	-0.046	0.968	0.767	0.936
Fcgr3	0.237	1.178	0.076	0.565
Fcgr4	0.406	1.325	0.741	0.927
Gpr34	0.594	1.509	0.050	0.526
Havcr2	-0.081	0.946	0.640	0.891
Ifngr1	0.448	1.364	0.145	0.641
Il10ra	0.131	1.095	0.650	0.895
Il21r	0.044	1.031	0.845	0.959
Il6ra	-0.698	0.617	0.129	0.626
Itgam	-0.198	0.872	0.740	0.926
Itgam	-2.090	0.235	0.381	0.785
Itgb2	-0.167	0.891	0.646	0.894
Lair1	1.727	3.310	0.019	0.456
Mpeg1	0.295	1.226	0.174	0.669
Ms4a6b	0.647	1.565	0.152	0.648
P2ry12	-0.537	0.689	0.141	0.638
P2ry13	-0.444	0.735	0.020	0.464
Parvg	-1.130	0.457	0.071	0.560
Ptafr	0.497	1.411	0.077	0.565
Ptprc	0.862	1.817	0.083	0.576
Selplg	-0.180	0.883	0.131	0.628
Slc11a1	0.123	1.089	0.437	0.817
Slc16a3	0.333	1.260	0.696	0.911
Slc2a5	-1.426	0.372	0.015	0.446
Slco2b1	-0.066	0.955	0.589	0.874
Stab1	-1.085	0.471	0.257	0.719
Tgfbr1	0.564	1.478	0.019	0.458
Tlr1	0.391	1.311	0.126	0.622
Tlr2	0.678	1.600	0.042	0.512
Tlr5	-1.404	0.378	0.096	0.593

Tlr6	0.168	1.123	0.570	0.868
Tlr7	-0.501	0.706	0.181	0.676
Tmem1 19	-0.143	0.905	0.634	0.889
Tnfrsf1b	-0.384	0.766	0.106	0.605
Trem2	1.496	2.820	0.064	0.552
**Chiu et al. D100**				
Gene name	Log2 fold change	Fold-change.Mutant/Control	P-value	FDR
Adora3	-0.123	0.918	0.540	0.776
Adrb2	0.264	1.201	0.335	0.641
C3ar1	0.802	1.744	0.001	0.033
C5ar1	1.191	2.283	0.021	0.165
Ccr5	-0.435	0.740	0.116	0.394
Cd14	1.642	3.120	0.001	0.039
Cd180	0.524	1.438	0.034	0.213
Cd300a	-0.717	0.608	0.004	0.066
Cd33	-0.066	0.955	0.789	0.912
Cd37	-0.705	0.613	0.132	0.419
Cd53	0.461	1.377	0.057	0.274
Cd68	1.476	2.782	0.000	0.020
Cd84	0.641	1.560	0.021	0.162
Cd86	0.462	1.378	0.083	0.333
Clec5a	1.538	2.904	0.000	0.016
Clec7a	4.413	21.299	0.000	0.010
Cmklr1	-0.321	0.801	0.167	0.470
Csf1r	-0.082	0.944	0.682	0.859
Csf2rb	0.233	1.175	0.114	0.389
Csf3r	-0.172	0.887	0.342	0.646
Cx3cr1	-0.296	0.814	0.260	0.576
Fcgr1	0.123	1.089	0.524	0.766
Fcgr3	0.297	1.229	0.190	0.499
Fcgr4	1.942	3.844	0.005	0.080
Gpr34	0.292	1.225	0.091	0.348
Havcr2	-0.270	0.830	0.269	0.584
Ifngr1	-0.115	0.923	0.541	0.776
Il10ra	-0.310	0.807	0.305	0.617

Il21r	-0.188	0.878	0.618	0.821
Il6ra	-0.607	0.656	0.013	0.129
Itgam	0.466	1.382	0.216	0.529
Itgam	-1.498	0.354	0.144	0.437
Itgb2	0.656	1.576	0.000	0.013
Lair1	-1.538	0.344	0.153	0.452
Mpeg1	1.898	3.728	0.000	0.005
Ms4a6b	-0.330	0.796	0.493	0.747
P2ry12	-0.680	0.624	0.003	0.055
P2ry13	-0.513	0.701	0.049	0.255
Parvg	0.373	1.295	0.063	0.289
Ptafr	0.133	1.096	0.719	0.881
Ptprc	1.112	2.162	0.000	0.008
Selplg	-0.621	0.650	0.040	0.230
Slc11a1	0.527	1.441	0.178	0.483
Slc16a3	0.760	1.693	0.476	0.736
Slc2a5	-1.594	0.331	0.018	0.148
Slco2b1	-0.177	0.885	0.457	0.725
Stab1	-1.142	0.453	0.079	0.326
Tgfbr1	0.025	1.018	0.882	0.954
Tlr1	0.512	1.426	0.008	0.100
Tlr2	2.249	4.754	0.000	0.012
Tlr5	-1.440	0.369	0.053	0.263
Tlr6	0.373	1.295	0.234	0.549
Tlr7	0.254	1.193	0.351	0.653
Tmem119	-0.903	0.535	0.020	0.159
Tnfrsf1b	-0.438	0.738	0.169	0.472
Trem2	0.649	1.568	0.082	0.331
**Chiu et al. D130**				
Gene name	Log2 fold change	Fold-change.Mutant/Control	P-value	FDR
Adora3	-0.341	0.790	0.180	0.462
Adrb2	-0.874	0.546	0.012	0.104
C3ar1	1.638	3.113	0.000	0.011
C5ar1	1.188	2.278	0.091	0.326
Ccr5	-1.187	0.439	0.000	0.003

Cd14	1.241	2.363	0.001	0.024
Cd180	0.704	1.629	0.005	0.058
Cd300a	-0.048	0.967	0.868	0.944
Cd33	-0.153	0.899	0.171	0.450
Cd37	0.120	1.087	0.821	0.922
Cd53	0.430	1.347	0.119	0.374
Cd68	1.866	3.644	0.000	0.003
Cd84	1.021	2.029	0.001	0.017
Cd86	0.712	1.638	0.002	0.034
Clec5a	0.099	1.071	0.657	0.841
Clec7a	5.153	35.590	0.000	0.004
Cmklr1	0.444	1.360	0.014	0.113
Csf1r	-0.233	0.851	0.532	0.767
Csf2rb	0.669	1.590	0.005	0.062
Csf3r	-0.003	0.998	0.977	0.990
Cx3cr1	-0.741	0.598	0.174	0.455
Fcgr1	-0.316	0.803	0.042	0.214
Fcgr3	-0.031	0.979	0.906	0.960
Fcgr4	2.032	4.089	0.000	0.013
Gpr34	-0.331	0.795	0.213	0.501
Havcr2	0.437	1.354	0.111	0.360
Ifngr1	-0.601	0.659	0.050	0.237
Il10ra	-0.601	0.659	0.001	0.026
Il21r	-0.173	0.887	0.757	0.891
Il6ra	-1.083	0.472	0.015	0.119
Itgam	0.260	1.197	0.575	0.793
Itgam	-0.393	0.761	0.608	0.813
Itgb2	1.176	2.259	0.000	0.010
Lair1	0.700	1.625	0.113	0.364
Mpeg1	1.537	2.902	0.000	0.003
Ms4a6b	0.132	1.096	0.597	0.807
P2ry12	-1.560	0.339	0.000	0.002
P2ry13	-1.278	0.412	0.000	0.001
Parvg	0.269	1.205	0.372	0.652
Ptafr	-0.029	0.980	0.925	0.968
Ptprc	1.674	3.191	0.005	0.058
Selplg	-1.199	0.435	0.002	0.034
Slc11a1	0.977	1.969	0.022	0.147
Slc16a3	1.704	3.257	0.018	0.131

Slc2a5	-2.730	0.151	0.001	0.020
Slco2b1	-1.080	0.473	0.000	0.004
Stab1	-1.070	0.476	0.001	0.028
Tgfbr1	0.193	1.143	0.273	0.564
Tlr1	0.739	1.669	0.002	0.038
Tlr2	2.376	5.191	0.003	0.042
Tlr5	-3.878	0.068	0.003	0.044
Tlr6	0.002	1.002	0.992	0.996
Tlr7	0.249	1.189	0.223	0.513
Tmem119	-1.242	0.423	0.000	0.005
Tnfrsf1b	0.117	1.085	0.432	0.698
Trem2	-0.745	0.597	0.513	0.756
**Galatro et al.**				
Gene name	logFC	P.Value		
CD180	-0.022	0.000		
CD53	-0.007	0.008		
IFNGR1	-0.013	0.002		
IL6R	-0.011	0.001		
LPAR5	-0.010	0.008		
P2RY12	-0.017	0.007		
CLEC5A	0.051	0.002		
MS4A6A	0.029	0.003		
**Holtman et al.**				
Gene Name	AGED_logFC	AGED_FDR_p	APP_logFC	APP_FDR_p
ADORA3	-0.226	0.141	-0.711	0.000
ADRB2	-0.078	0.436	-0.684	0.000
C3AR1	0.126	0.179	0.122	0.087
C5AR1	0.017	0.743	0.565	0.005
CCR5	-0.120	0.083	-0.924	0.000
CD14	-0.024	0.913	2.254	0.000
CD180	0.347	0.022	0.167	0.435
CD300A	-0.021	0.945	-0.523	0.016

CD33	-0.020	0.889	-0.631	0.000
CD37	-0.009	0.967	-0.007	0.983
CD53	-0.056	0.386	-0.050	0.724
CD68	0.045	0.789	0.889	0.000
CD84	0.187	0.308	0.595	0.000
CD86	-0.098	0.445	-0.049	0.808
CLEC5A	0.010	0.960	0.029	0.882
CLEC7A	1.809	0.000	2.949	0.000
CMKLR1	-0.034	0.584	-0.187	0.208
CSF1R	-0.180	0.305	-1.072	0.000
CSF2RB	0.076	0.489	0.136	0.266
CSF3R	-0.127	0.557	-0.450	0.048
CX3CR1	-0.119	0.383	-1.053	0.000
GPR34	-0.248	0.071	-0.553	0.023
HAVCR2	0.041	0.527	-0.228	0.249
IFNGR1	-0.480	0.061	-0.821	0.000
IL10RA	-0.274	0.112	-0.080	0.629
IL21R	-0.063	0.514	-0.458	0.008
ITGAM	0.069	0.302	-0.491	0.000
ITGB2	0.324	0.113	-0.174	0.099
LAIR1	-0.012	0.935	-0.197	0.152
P2RY12	-0.191	0.000	-0.962	0.001
P2RY13	-0.165	0.075	-0.888	0.000
PARVG	-0.116	0.629	-0.313	0.026
PTPRC	0.160	0.062	0.449	0.104
SELPLG	-0.200	0.030	-1.215	0.000
SLC11A1	0.278	0.131	0.473	0.000
SLC16A3	0.093	0.719	2.206	0.000
SLC2A5	-0.126	0.430	-1.149	0.000
SLCO2B1	-0.197	0.000	-0.710	0.010
STAB1	-0.271	0.372	-1.177	0.000
TGFBR1	-0.218	0.001	-0.560	0.094
TLR1	0.180	0.385	0.154	0.319
TLR2	0.822	0.000	1.607	0.000

TLR6	0.075	0.750	0.147	0.543
TLR7	0.002	0.994	-0.152	0.449
TMEM119	-0.129	0.299	-1.412	0.000
TNFRSF1B	-0.028	0.715	-0.338	0.003
TREM2	-0.026	0.934	0.751	0.000
**Holtman et al.**				
Gene Name	ERCC1_logFC	ERCC1_FDR_p	SOD1_logFC	SOD1_FDR_p
ADORA3	-0.028	0.939	-0.846	0.404
ADRB2	-0.214	0.016	-0.464	0.687
C3AR1	0.307	0.005	1.093	0.066
C5AR1	-0.400	0.017	1.623	0.009
CCR5	-0.404	0.008	-1.260	0.005
CD14	-0.108	0.570	0.688	0.533
CD180	0.275	0.278	0.763	0.447
CD300A	-0.092	0.611	0.254	0.774
CD33	-0.160	0.128	0.189	0.833
CD37	-0.027	0.935	0.244	0.898
CD53	-0.159	0.029	0.163	0.898
CD68	0.345	0.004	1.769	0.008
CD84	0.483	0.000	0.233	0.777
CD86	0.203	0.022	0.652	0.513
CLEC5A	0.079	0.698	-0.006	1.000
CLEC7A	2.841	0.000	2.709	0.000
CMKLR1	0.137	0.756	0.179	0.873
CSF1R	-0.086	0.476	-0.323	0.775
CSF2RB	0.337	0.026	0.217	0.849
CSF3R	0.221	0.347	-0.045	0.991
CX3CR1	-0.323	0.001	-0.499	0.478
GPR34	-0.384	0.013	-0.548	0.525
HAVCR2	0.038	0.806	0.278	0.766
IFNGR1	-0.134	0.299	-0.462	0.587
IL10RA	-0.109	0.474	-0.550	0.507

IL21R	0.130	0.670	0.301	0.837
ITGAM	-0.171	0.681	0.310	0.668
ITGB2	0.224	0.016	1.110	0.183
LAIR1	-0.014	0.955	-0.177	0.865
P2RY12	-0.320	0.000	-1.398	0.007
P2RY13	-0.327	0.004	-1.321	0.005
PARVG	0.021	0.943	0.388	0.796
PTPRC	0.163	0.149	0.696	0.303
SELPLG	-0.191	0.051	-0.965	0.091
SLC11A1	-0.004	0.990	0.457	0.632
SLC16A3	0.081	0.530	1.812	0.105
SLC2A5	-0.794	0.000	-2.009	0.071
SLCO2B1	-0.221	0.001	-1.089	0.039
STAB1	-0.090	0.739	-0.745	0.433
TGFBR1	-0.249	0.000	-0.016	1.000
TLR1	0.493	0.001	0.281	0.837
TLR2	0.971	0.000	1.899	0.016
TLR6	0.259	0.256	-0.139	0.956
TLR7	-0.005	0.993	0.008	1.000
TMEM119	-0.123	0.245	-1.332	0.003
TNFRSF1B	0.047	0.874	-0.107	0.932
TREM2	0.161	0.233	-0.006	1.000

Keren Shaul et al. Homeostatic vs DAM

Gene Name	Fold-change.(DAM.to.homeostatic.microglia)	-log10(DAM).p-value.(Mann-Whitney)
Adora3	-1.677	3.621
Adrb2	-2.045	4.560
C3ar1	0.529	4.164
C5ar1	0.500	0.953
Ccr5	-2.341	22.334
Cd14	0.180	0.090
Cd180	1.147	8.198

Cd300a	-1.025	2.757
Cd33	-1.369	2.856
Cd37	0.360	2.684
Cd53	-0.237	1.062
Cd68	1.339	28.333
Cd84	0.400	3.895
Cd86	-0.272	0.262
Clec5a	-0.008	0.360
Clec7a	5.149	43.267
Cmklr1	-0.474	0.491
Csf1r	-0.469	15.545
Csf2rb	-0.512	0.405
Csf3r	0.728	3.529
Cx3cr1	-1.360	55.106
Fcgr1	-0.974	4.977
Fcgr3	0.192	0.444
Fcgr4	1.998	4.155
Gpr183	-0.902	2.292
Gpr34	-0.858	12.216
Havcr2	0.194	1.249
Ifngr1	-1.587	13.061
Il10ra	-1.277	8.879
Il21r	-1.872	3.148
Il6ra	-1.789	10.210
Itgam	-1.124	11.222
Itgb2	0.724	6.755
Lair1	-0.693	2.277
Mpeg1	1.307	30.324
Ms4a6b	-2.028	4.882
P2ry12	-2.052	61.169
P2ry13	-1.587	23.445
Parvg	-0.130	0.640
Ptafr	-1.238	4.631
Ptprc	0.311	2.200
Selplg	-1.225	39.414
Slc11a1	0.486	3.854
Slc16a3	1.120	3.420
Slc2a5	-1.511	5.188
Slco2b1	-1.270	17.936

Stab1	-2.526	8.737	
Tgfbr1	-0.674	8.196	
Tlr1	-0.569	0.334	
Tlr2	0.924	3.460	
Tlr7	-0.270	1.422	
Tmem119	-2.456	58.756	
Tnfrsf1b	-1.595	5.833	
Trem2	1.227	29.378	
**Olah et al.**			
Gene Name	logFC	P.Value	adj.P.Val
ADORA3	-1.062	0.006	0.017
ADRB2	-0.220	0.816	0.876
C3AR1	-2.240	0.000	0.000
C5AR1	-4.662	0.000	0.000
CCR5	-3.336	0.000	0.000
CD14	5.494	0.000	0.000
CD180	-1.435	0.052	0.101
CD300A	1.385	0.007	0.020
CD33	0.706	0.104	0.179
CD37	4.413	0.000	0.000
CD53	-1.072	0.001	0.002
CD68	1.775	0.000	0.001
CD84	-0.319	0.585	0.690
CD86	-1.234	0.005	0.015
CLEC5A	-0.971	0.392	0.507
CLEC7A	-2.325	0.000	0.000
CMKLR1	-0.052	0.904	0.938
CSF1R	1.642	0.003	0.009
CSF2RB	0.240	0.692	0.782
CSF3R	3.357	0.000	0.000
CX3CR1	-1.941	0.006	0.017
FCGR1A	1.135	0.099	0.172

FCGR2A	-1.054	0.022	0.051
FCGR3A	0.969	0.041	0.084
GPR183	-4.013	0.000	0.000
GPR34	-1.199	0.019	0.044
HAVCR2	-1.696	0.000	0.000
IFNGR1	-0.810	0.109	0.187
IL10RA	0.519	0.314	0.429
IL6R	-1.576	0.004	0.013
ITGAM	-0.305	0.490	0.603
ITGB2	2.669	0.000	0.000
LAIR1	2.677	0.000	0.000
LPAR5	0.347	0.453	0.569
MPEG1	-4.818	0.000	0.000
MS4A6A	2.140	0.000	0.002
P2RY12	-2.801	0.000	0.000
P2RY13	-2.925	0.000	0.000
PARVG	2.065	0.000	0.002
PTAFR	1.762	0.004	0.012
PTPRC	-0.767	0.161	0.255
SELPLG	2.350	0.002	0.008
SLC11A1	3.243	0.001	0.003
SLC16A3	3.897	0.000	0.000
SLC2A5	0.947	0.031	0.067
SLCO2B1	2.146	0.002	0.006
STAB1	4.420	0.000	0.000
TGFBR1	-1.828	0.000	0.002
TLR1	-1.133	0.042	0.085
TLR2	-2.625	0.000	0.001
TLR5	0.757	0.245	0.354
TLR6	-1.884	0.002	0.006
TLR7	-0.718	0.108	0.185
TMEM119	2.050	0.000	0.001
TNFRSF1B	0.071	0.914	0.945

TREM2	2.015	0.001	0.002	
**Tay et al.**				
Gene Name	FNX_log2foldchange	FNX_adjusted_pvalues	FAD_log2foldchange	FAD_adjusted_pvalues
Adora3	0.228	1.000	-1.677	0.003
Adrb2	-0.643	1.000	-2.045	0.000
C3ar1	-0.381	1.000	0.529	0.001
C5ar1	0.047	1.000	0.500	0.343
Ccr5	-0.289	1.000	-2.341	0.000
Cd14	-0.479	0.581	0.180	0.925
Cd180	0.207	1.000	1.147	0.000
Cd300a	-0.352	1.000	-1.025	0.015
Cd33	0.162	1.000	-1.369	0.012
Cd37	-0.016	1.000	0.360	0.017
Cd53	-0.052	1.000	-0.237	0.287
Cd68	-0.024	1.000	1.339	0.000
Cd84	-0.015	1.000	0.400	0.002
Cd86	-0.093	1.000	-0.272	0.786
Clec5a	0.088	1.000	-0.008	0.719
Clec7a	0.366	1.000	5.149	0.000
Cmklr1	-0.257	1.000	-0.474	0.613
Csf1r	-0.069	1.000	-0.469	0.000
Csf2rb	0.274	1.000	-0.512	0.682
Csf3r	0.149	1.000	0.728	0.003
Cx3cr1	-0.316	0.020	-1.360	0.000
Fcgr1	0.383	0.392	-0.974	0.000
Fcgr3	-0.196	1.000	0.192	0.653
Fcgr4	1.165	0.009	1.998	0.001
Gpr183	-0.430	1.000	-0.902	0.037
Gpr34	-0.912	0.000	-0.858	0.000
Havcr2	-0.322	1.000	0.194	0.213
Ifngr1	-0.477	0.318	-1.587	0.000
Il10ra	0.247	1.000	-1.277	0.000
Il21r	0.084	1.000	-1.872	0.007
Il6ra	-0.185	1.000	-1.789	0.000
Itgam	0.758	0.000	-1.124	0.000
Itgb2	-0.111	1.000	0.724	0.000
Lair1	-0.173	1.000	-0.693	0.038

Mpeg1	0.559	0.000	1.307	0.000
Ms4a6b	-0.314	1.000	-2.028	0.000
P2ry12	-0.841	0.000	-2.052	0.000
P2ry13	-0.605	0.004	-1.587	0.000
Parvg	-0.165	1.000	-0.130	0.516
Ptafr	0.076	1.000	-1.238	0.000
Ptprc	0.134	1.000	0.311	0.043
Selplg	-0.506	0.000	-1.225	0.000
Slc11a1	0.089	1.000	0.486	0.002
Slc16a3	-0.007	1.000	1.120	0.004
Slc2a5	-0.845	0.012	-1.511	0.000
Slco2b1	-0.261	1.000	-1.270	0.000
Stab1	-0.869	0.063	-2.526	0.000
Tgfbr1	-0.576	0.001	-0.674	0.000
Tlr1	0.454	1.000	-0.569	0.732
Tlr2	0.147	1.000	0.924	0.004
Tlr7	0.154	1.000	-0.270	0.164
Tmem119	-0.309	0.216	-2.456	0.000
Tnfrsf1b	-0.224	1.000	-1.595	0.000
Trem2	0.140	1.000	1.227	0.000

Tay et al.

Gene Name	CK-p25_log2foldchange	CK-p25_adjusted_pvalues
Adora3	-0.329	0.319
Adrb2	-2.332	0.000
C3ar1	0.777	0.000
C5ar1	0.239	0.668
Ccr5	-1.226	0.000
Cd14	0.239	0.416
Cd180	0.688	0.000
Cd300a	-0.508	0.001
Cd33	-0.419	0.002
Cd37	-0.060	0.900
Cd53	-0.449	0.000
Cd68	0.508	0.000
Cd84	0.568	0.000

Cd86	0.568	0.004
Clec5a	0.419	0.236
Clec7a	5.143	0.000
Cmklr1	-0.150	0.723
Csf1r	0.000	1.000
Csf2rb	0.478	0.030
Csf3r	0.658	0.000
Cx3cr1	0.000	0.828
Fcgr1	0.628	0.000
Fcgr3	-0.030	1.000
Fcgr4	2.960	0.000
Gpr183	-0.867	0.000
Gpr34	-1.256	0.000
Havcr2	0.120	0.777
Ifngr1	-1.077	0.000
Il10ra	-0.209	0.340
Il21r	0.120	0.833
Il6ra	-0.867	0.000
Itgam	-0.120	0.615
Itgb2	0.867	0.000
Lair1	0.060	0.819
Mpeg1	1.136	0.000
Ms4a6b	-0.299	0.515
P2ry12	-1.405	0.000
P2ry13	-1.435	0.000
Parvg	-0.239	0.464
Ptafr	0.030	0.999
Ptprc	0.718	0.000
Selplg	-1.316	0.000
Slc11a1	0.957	0.000
Slc16a3	0.628	0.194
Slc2a5	-2.063	0.000
Slco2b1	-0.777	0.000
Stab1	-1.495	0.000
Tgfbr1	-0.568	0.000
Tlr1	1.615	0.000
Tlr2	2.422	0.000
Tlr7	0.120	0.782

Tmem1 19	-1.376	0.000
Tnfrsf1b	-0.568	0.005
Trem2	0.449	0.000
